# Molecular Acquisition, Cleaning and Evaluation in R (MACER) - A tool to assemble molecular marker datasets from BOLD and GenBank

**DOI:** 10.3897/BDJ.9.e71378

**Published:** 2021-09-08

**Authors:** Robert G Young, Rekkab Gill, Daniel Gillis, Robert H Hanner

**Affiliations:** 1 University of Guelph, Guelph, Canada University of Guelph Guelph Canada

**Keywords:** DNA barcode, molecular marker, metabarcode, haplotype, database, R package

## Abstract

Molecular sequence data is an essential component for many biological fields of study. The strength of these data is in their ability to be centralised and compared across research studies. There are many online repositories for molecular sequence data, some of which are very large accumulations of varying data types like NCBI’s GenBank. Due to the size and the complexity of the data in these repositories, challenges arise in searching for data of interest. While data repositories exist for molecular markers, taxa and other specific research interests, repositories may not contain, or be suitable for, more specific applications. Manually accessing, searching, downloading, accumulating, dereplicating and cleaning data to construct project-specific datasets is time-consuming. In addition, the manual assembly of datasets presents challenges with reproducibility. Here, we present the MACER package to assist researchers in assembling molecular datasets and provide reproducibility in the process.

## Introduction

The use of molecular sequence data is an essential component of many analyses across various fields of scientific study, including virology (Radford et al. 2012), gene expression studies (Song et al. 2021), evolutionary biology (Hudson 2008), taxonomy and species identifications (Hubert and Hanner 2015) and biodiversity surveys (Young et al. 2021). One of the strengths of molecular approaches is in the comparability of the data, even when obtained by different labs and researchers. To support research efforts, there are numerous database resources to house molecular sequence data. These resources are essential to aggregate data for scientific research, provide a repository for transparent publishing and reproducibility and act as a data source for big data studies. Three of the main molecular sequence resources are the USA National Center for Biotechnology Information (NCBI) database GenBank (Benson et al. 2018; https://www.ncbi.nlm.nih.gov/genbank/), the European Molecular Biology Laboratory (EMBL) database ENSEMBL (Baker et al. 2000; http://www.ensembl.org/) and Japan’s National Institute of Genetics DNA Data Bank of Japan (DDBJ; Bower 1989, Kaminuma et al. 2009, Mashima et al. 2016; https://www.ddbj.nig.ac.jp/about/index-e.html).

These three data resources exchange information through the International Nucleotide Sequence Database Collaboration (INSDC) storage and sharing agreement (www.insdc.org). INSDC establishes basic standards for upload; however, these standards are not always sufficient for studies requiring robust metadata associated with molecular records. Furthermore, the INSDC databases are simply a repository and lack ongoing curation and maintenance of records. Another challenge is that, with the inclusion of a wide range of molecular data from various molecular research efforts, the extensive size of these databases makes it challenging to manually assemble specific datasets.

Due to these challenges, more focused molecular sequence repositories have been established. These data resources often focus on targeted efforts for assembling molecular evidence specific to taxa, molecular marker or stated research goals. There are over 1,700 specific data resources as of 2018 (Imker 2018) having numerous foci with respect to molecular biological databases, including expression databases, protein structure and nucleotide sequence data (Drysdale et al. 2020). Here, we focus on the use of molecular sequence data for species identification and uses in biodiversity surveys.

Focused molecular sequence repositories obtain data through direct submission and through scraping the INSDC databases. Often the direct submission of records to these databases have stricter quality controls for upload of data. Databases such as the UNITE database (Abarenkov et al. 2010, Nilsson et al. 2018; https://unite.ut.ee/) for fungal identifications and the Silva database (Quast et al. 2012; https://www.arb-silva.de/) with ribosomal sequences for all domains of life include quality control checks and analytical options associated with their repositories. The Barcode of Life Data system (BOLD; Ratnasingham and Hebert 2007; http://www.boldsystems.org/) is amongst the larger of these focused repositories and is largely populated by cytochrome *c* oxidase subunit I (COI-5P) molecular data.

The use of focused molecular sequence databases do have advantages over the larger INSDC resources, but there are also challenges. When addressing specific research questions, some focused data resources may be too restrictive or not limited enough. Another factor may be the need to assemble smaller taxonomically focused datasets useful for informatics on local infrastructure for easier customisation and faster analyses. Additionally, static datasets are necessary for reproducibility as opposed to using regularly changing databases with ongoing additions or curation efforts. Custom static datasets are especially important for regulatory studies where outcomes from an investigation need to be applied in a legal framework or regulated through an overseeing body or government. In these situations, the custom assembly of nucleotide sequence datasets is needed. In addition, beyond simple assembly, the cleaning of records from INSDC and other databases like BOLD, is essential to ensure high quality data. The method by which the cleaning of these datasets is completed is essential for good scientific practices. Often cleaning of molecular sequence datasets is described as ‘manually edited’ and this is concerning as there is no reproducibility to this method.

Here, we present the Molecular Acquisition, Cleaning and Evaluation in R (MACER) package for the R statistical computing and graphics environment (R Core Team 2020, version 4.1.0). The package was tested using a Windows 10 x64 operating system and a macOS Big Sur 10.16 operating system prior to submission to CRAN. MACER accepts a list of genera as a user input and uses NCBI-GenBank and BOLD as resources to download and assemble molecular sequence datasets. These datasets are then assembled by marker, aligned, trimmed and cleaned. The use of this package allows the publication of specific parameters to ensure reproducibility. The MACER package has four core functions that are described below and an example can be accessed through the online repositories (https://github.com/rgyoung6/MACER and https://github.com/rgyoung6/MACER_example).

## Installation

Package MACER is available on CRAN and can be downloaded there. See: https://CRAN.R-project.org/package=MACER. The development version is available on GitHub as well. See: https://github.com/rgyoung6/MACER.

# Install from CRAN

install.packages('MACER’)

# Install development version from GitHub

devtools::install_github('rgyoung6/MACER')

In addition, the MAFFT programme (external to R and MACER) needs to be installed (Katoh and Standley 2013; https://mafft.cbrc.jp/alignment/software/)

## Dependencies

There are three R language dependencies and one external dependency associated with the MACER package. The reentrez package (ver. 1.2.3; Winter 2017) is used to access and download data from the NCBI GenBank servers. The httr package is used in error handling with web connections (ver. 1.4.2; Wickham 2020). The ape package (ver. 5.5; Paradis and Schliep 2019) is used to construct a pairwise distance matrix and assess genus level and species level statistical outliers. In addition, the MAFFT alignment programme is utilised to align downloaded sequences against the reference sequence (Katoh and Standley 2013; https://mafft.cbrc.jp/alignment/software/). Two other core associated packages are also listed as dependencies including stats and utils (R Core Team 2020). Finally, while not a dependency, the documentation of the package accompanying this publication was created through the use of roxygen2 (ver. 7.1.1; Wickham et al. 2020).

## Core Functions

There are four functions associated with the package. The first is the sequence download function, *auto_seq_download()*, which takes a user-supplied list of target genera and downloads data from GenBank and BOLD or either individually. The second function, *create_fastas()*, takes a user-supplied table of genera of interest and target molecular marker names to assemble taxa and marker-specific FASTA files. This step is necessary due to the inconsistency in naming conventions and presence of typographical errors in databases (common amongst GenBank records). Once assembled, there is a function for alignment and trimming of the target dataset, *align_to_ref()*. This alignment and trimming uses an installed version of MAFFT on the local computer and a reference sequence for the target molecular region. The final function, *barcode_clean()*, analyses the output data and cleans, based on user-selected elements including amino acid translation and presence of non-AGCT characters. The strength of this four-step approach is in the ability to exactly replicate the download, alignment and cleaning of a dataset and support reproducibility in science, while, at the same time, providing an easy implementation to obtain molecular sequence data necessary for studies.

***auto_seq_download()***

This is a function to download marker DNA sequences from both BOLD and GenBank. The input for this function is a file containing a list of genera you want to download in a single column with an empty line at the bottom of the list.

**Arguments**

BOLD_database – TRUE is to include, FALSE is to exclude; default TRUE

NCBI_database – TRUE is to include, FALSE is to exclude; default TRUE

search_str – NULL uses the default string; anything other than NULL, then that string will be used for the GenBank search; default NULL. The default string is: *(genus[ORGN]) NOT (shotgun[ALL] OR genome[ALL] OR assembled[ALL] OR microsatellite[ALL])*.

input_file – NULL prompts the user to indicate the location of the input file through point and click prompts; anything other than NULL, then the string supplied will be used for the location; default NULL

output_file – NULL prompts the user to indicate the location of the output file through point and click prompts; anything other than NULL, then the string supplied will be used for the location; default NULL

Note: When using a custom search string for NCBI, only a single genus at a time can be searched.

**Output**

The output from this function is located in a single main file folder which contains three sub-folders with file described below.

Main folder - Seq_auto_dl_TTTTTT_MMM_DD

BOLD - Contains a file for every genus downloaded with the raw data from BOLD.

NCBI - Contains a file for every genus downloaded with the raw data from GenBank.

Total_tables - Contains data obtained from running of the function.

*A_Summary.txt* - Information about the download process for each genus including species and molecular markers.

*A_Total_Table.dat* - A single table containing the accumulated data for all genera searched.

**Dependencies**

The function uses rentrez (ver. 1.2.3; Winter 2017) to access and download sequences from NCBI’s GenBank and this is required to run the function.

***create_fastas()***

This is a function that uses the *A_Total_Table.dat* from the *auto_seq_download()* and an additional parameter file to place records into FASTA files specific to genus and molecular marker. The parameter file contains a list of genera with the molecular markers names below the taxa names. The information to create this parameter file can be obtained from *A_Summary.txt* file from the *auto_seq_download()* output (see Table 1).

**Arguments**

data_file – NULL prompts the user to indicate the location of the data file in the format of the auto_seq_download output; anything other than NULL, then the string supplied will be used for the location; default NULL

input_file – NULL prompts the user to indicate the location of the input file used to select through point and click prompts; anything other than NULL, then the string supplied will be used for the location; default NULL

output_folder – NULL prompts the user to indicate the location of the output file through point and click prompts; anything other than NULL, then the string supplied will be used for the location; default NULL

no_marker – If set to TRUE, then records will be included if flagged due to no marker data. Default is FALSE to not include records with no marker data.

no_taxa – If set to TRUE, then records will be included if flagged due to no taxa data. Default is FALSE to not include records with no taxa data.

no_seq – If set to TRUE, then records will be included if flagged due to no sequence data. Default is FALSE to not include records with no sequence data.

name_issue – If set to TRUE, then records will be included if flagged due to genus and species names with more than two terms. Default is FALSE to not include records with taxonomic naming issues.

taxa_digits – If set to TRUE, then included if flagged due to genus or species names containing digits. Default is FALSE to not include records with digits in the taxonomic naming.

taxa_punct – If set to TRUE, then records will be included if flagged due to the presence of punctuation in the genus or species names. Default is FALSE to not include records with punctuation in the taxonomic naming.

**Output**

This function outputs a FASTA file of sequences for each column in the submitted parameters file. These files are named with the genera of interest and the first marker name in the column of the parameters file. These files are located in the folder where the *Total_tables.dat* file is located.

**Dependencies**

There are no dependencies for this function.

***align_to_ref()***

This function takes FASTA files with target sequences and aligns them against a reference sequence submitted to the programme. The function requests a file folder location with the FASTA files of interest upon execution. It also requests the location of a single sequence in a FASTA file format. Finally, the function requests the folder with the MAFFT executable. All files in the folder are then aligned one at a time to the reference sequence. For each file, the aligned output is trimmed to the length of the target sequence and sequences without full coverage (records having sequences with leading or trailing gaps) are removed. Finally, internal gaps are removed, if indicated, from the sequence. This removal is based on the submitted multiple sequence alignment percent internal gap coverage of the character position as provided in the pigl argument supplied by the user. The function also accepts a gap opening penalty argument.

**Arguments**

data_folder – This variable can be used to provide a location for the file containing all of the fasta files wanting to be aligned. The default value is set to NULL where the programme will prompt the user to select the folder through point-and-click.

ref_seq_file – This variable can be used to provide a location for the reference sequence file. The default value is set to NULL where the programme will prompt the user to select the folder through point-and-click.

MAFFT_loc – This variable can be used to provide a location for the MAFFT programme. The default value is set to NULL where the programme will prompt the user to select the folder through point-and-click.

output_file – This variable can be used to set the location of the output files from the programme. The default value is set to NULL where the programme will place the output files in the same location as the target files.

pigl – This is the proportion internal gap loop argument. This provides a proportion threshold that will trigger the removal of records causing internal gaps if more than the proportional value assigned to this argument is reached. If this value is set to 0, then internal gaps are not removed. The default for this value is 0.95.

op – This is the gap opening penalty for the use of MAFFT. The higher the value, the larger the penalty in the alignment. The default for this value is set to 10. The default value in the MAFFT programme is 1.53. For alignment of highly conserved regions where no gaps are expected, this should be set to a much higher number and 10 is recommended for barcode regions like the COI-5P.

**Output**

This function outputs a log file, *MAFFT_log.txt* and file folders, MAFFT and MAFFT_trimmed in the same location as the target FASTA files of interest. The MAFFT and the MAFFT_trimmed folders each contain a file for each submitted FASTA file of interest. In the MAFFT folder, there will be files with the names of each of the target files appended with ‘*_MAFFT.fas*’. The same is true for the aligned and trimmed files, but appended with ‘*_MAFFT_trimmed.fas*’. When viewing the multiple sequence alignment files, if there are gaps in the alignment, a user can increase the op value in an attempt to force an alignment with fewer gaps. If this still does not solve the issue of a gappy alignment, manually investigating the alignment for non-target taxonomic records should be conducted. Finally, if both of these measures are not sufficient to enable an alignment with few gaps, where few gaps are expected to be present, please investigate the MAFFT documentation to address your concerns.

**Dependencies**

While not an R dependency, the MAFFT programme must be installed on the user's computer (Katoh and Standley 2013; https://mafft.cbrc.jp/alignment/software/).

***barcode_clean()***

This function takes a trimmed multiple sequence alignment in FASTA file format and removes genus-level outliers and species outliers. To identify outliers, a pairwise p-distance matrix is calculated. Sequences that are greater than 1.5x pairwise sequence distance are identified as outliers. This pairwise sequence distance is evaluated at both the genus and species levels to identify and filter out outliers. *barcode_clean()* also, if selected, verifies the sequence using amino acid translation and/or eliminates sequences that have non-IUPAC codes. Finally, the programme calculates the barcode gap for the species in the submitted dataset.

**Arguments**

AA_code – This is the amino acid translation matrix used to check the sequences for stop codons. Assigning the AA_code to FALSE skips this part of the function. This assigns the AA_code to "std" for the standard translation amino acid code, "vert" for the vertebrate mitochondrial and "invert" for the invertebrate mitochondrial. The invertebrate mitochondrial matrix is the default.

AGCT_only – When this argument is set to TURE, the function only keeps sequences with AGCT exclusively and not other IUPAC characters. When set to FALSE, all IUPAC characters are accepted. The default is TRUE.

data_folder – This variable can be used to provide a location for the MSA FASTA files to be cleaned. The default value is set to NULL where the programme will prompt the user to select the folder through a point-and-click prompt.

**Output**

A single log file for the running of the function with the name A_Clean_File_YYYY-DD-TTTTTTTT. The function will also output three files for each FASTA file submitted. The first is the distance matrix that was calculated and used to assess the DNA barcode gaps. This file is named the same as the input file with ‘*_dist_table.dat*’ appended to the end of the name. The second file is the total data table file which provides a table of all submitted records for each dataset, accompanied by the results from each section of the analysis. This file is named the same as the input FASTA with ‘*_data_table.dat*’ appended to the end. Finally, a FASTA file with all outliers and flagged records removed is generated for each input FASTA file. This output file is named the same as the input FASTA with ‘*_no_outlier.fas*’ appended to the end. The flags that are possible are non_AGCT, Stop_Codon, Genus_Outlier, Species_Outlier and '-'.

**Dependencies**

The function was built using ape 5.4-1 and ape is required for distance matrix construction.

## Discussion

The construction of project-specific datasets is a challenge due to the time-consuming nature of the process and the reproducibility of the methods. Utilising automated processes, like MACER, to initially construct datasets for use in downstream analyses provides an efficient and reproducible method for research studies. There are three central strengths to the MACER approach. The first is in the standard input and output options, the second is the open-source nature of the tool and the third is the extensibility of the programme.

The MACER package requires the input of user data and arguments at several points for the four main functions. These inputs are short onscreen arguments, a simple list, a table of several lists or data in standard formats (e.g. FASTA). All lists are stored in flat files and not tied to any particular proprietary programme for long term access in adherence with the FAIR Data Principles (Findable, Accessible, Interoperable and Reusable; Wilkinson et al. 2016). The arguments used in the programme are easily included in published manuscripts to provide information for reusability in adherence with FAIR principles. Finally, aside from lists and arguments, the other data input format used in MACER is the universally-used nucleotide sequence data FASTA format (Pearson and Lipman 1988). Once a dataset has been obtained using MACER, additional analyses completed can also be recorded to adhere to FAIR principles.

The assembly of a molecular sequence dataset, through MACER or otherwise, is not a scientifically-important result. The ability and ease at which subsequent analyses are completed, using these targeted and clean sequence datasets, is paramount. The strength of the MACER approach is in the open-source package so that users can access and fully understand what is occurring when the programme is run. This open-source nature and the use of R is also ideal for these applications as it is a freely available open-source language which is extensively used in biological science research with a number of other informatics resources to support molecular sequence analyses (Gentleman et al. 2004, Brown et al. 2012, Paradis and Schliep 2019, Young et al. 2020). With access to the underlying code and the presence of R molecular sequence analysis tools, MACER is ideally suited to be included in analysis pipelines.

Finally, also due to its open-source nature, the MACER package is well suited for customisation and extension by users. With the MACER code structured in a modular format, there are opportunities to add functions and increase the analytical possibilities of the package. For instance, at present, MACER downloads and formats data from BOLD and GenBank. However, a function could be written to access data from other databases. Then, with the addition of a call to this function added in the main *auto_seq_download()* file, the number of data sources could be extended. Similarly, an alternative *align_to_ref()* could be written, using the current function as a model, utilising a different alignment algorithm, while keeping it incorporated into the current workflow. Finally, while the *barcode_clean()* function does currently assess the data, based on a number of metrics, including statistical outliers and amino acid translation, there are additional methods for assessing molecular sequence data and flagging potentially inaccurate data points (Zhang et al. 2012, Nugent and Adamowicz 2020, Fontes et al. 2021). With some of these being built in R, the integration of these additional cleaning and verification methods can be placed into the MACER pipeline.

## Citation, Web location (URIs) and repository

Researchers, using MACER for publications, should cite this article. Updated citation information can be obtained by typing citation("MACER") in R.

https://CRAN.R-project.org/package=MACER

https://github.com/rgyoung6/MACER

## Usage rights

It is open-source software (published under the GPL public licence, ver. 3).

## Figures and Tables

**Table 1.** An example of the file layout required for the parameter file for the *create_fastas()* function. The target genus is indicated at the top of each column. Each column represents the unique naming conventions for the target molecular marker. The data acquired for each column are then placed into a uniquely named FASTA file.

Neotamias	Neotamias	Neotamias
CYTB	COI-5P	18SRIBOSOMALRNA
CYTOCHROMEB	CYTOCHROMEBOXIDASE	18SSMALLSUBUNITRIBOSOMALRNA
CYTOCHROME-B	CYTOCHROMECOXIDASESUBUNIT1	
	CYTOCHROMECOXIDASESUBUNITI	
	CYTOCHROMEOXIDASESUBUNIT1	
	CYTOCHROMEOXIDASESUBUNITI	
